# Management of broncho-esophageal fistula after button battery ingestion

**DOI:** 10.1093/jscr/rjab441

**Published:** 2021-10-12

**Authors:** Katherine C Ott, Jamie C Harris, Katherine A Barsness, Jesse Arseneau, Saied Ghadersohi, Mehul V Raval

**Affiliations:** Division of Pediatric Surgery, Department of Surgery, Northwestern University Feinberg School of Medicine, Ann and Robert H. Lurie Children’s Hospital, Chicago, IL, USA; Division of Pediatric Surgery, Department of Surgery, Northwestern University Feinberg School of Medicine, Ann and Robert H. Lurie Children’s Hospital, Chicago, IL, USA; Division of Pediatric Surgery, Department of Surgery, Baylor College of Medicine, Children’s Hospital of San Antonio, CHRISTUS Health, San Antonio, TX, USA; Division of Pediatric Surgery, Department of Surgery, Northwestern University Feinberg School of Medicine, Ann and Robert H. Lurie Children’s Hospital, Chicago, IL, USA; Division of Otolaryngology–Head and Neck Surgery, Ann and Robert H. Lurie Children’s Hospital, Chicago, IL, USA; Division of Pediatric Surgery, Department of Surgery, Northwestern University Feinberg School of Medicine, Ann and Robert H. Lurie Children’s Hospital, Chicago, IL, USA

## Abstract

Button battery ingestion can cause serious injury or death in young children who cannot communicate symptoms. An 18-month-old male presented after his mother noted drooling, nonbilious emesis and a metallic smell to his breath. He underwent rigid esophagoscopy and a 3-V 20-mm button battery was removed. Subsequent bronchoscopy after a 1-week interval revealed progression to a large broncho-esophageal fistula on the posterior wall of the right mainstem bronchus past the carina. A fenestrated nasogastric tube for local control of secretion and a feeding jejunostomy was placed. Six weeks later, the patient underwent a right thoracotomy for division and repair of the fistula and intercostal muscle flap interposition. Utilizing a well-placed fenestrated nasogastric tube to manage secretions can help reduce fistula size and improve conservative management results. When surgical repair is required, an intercostal muscle flap can reinforce fistula closure while simultaneously buttressing the bronchus and esophagus.

## INTRODUCTION

The morbidity and mortality of button battery ingestions has risen in the last two decades as larger, higher voltage lithium batteries have become available [1, 2]. Both the North American Society for Pediatric Gastroenterology, Hepatology & Nutrition (NASPGHAN) and the European Society for Paediatric Gastroenterology Hepatology and Nutrition (ESPGHAN) recommend emergent endoscopic removal of button batteries in the esophagus [3, 4]. Many tertiary centers have created protocols involving activation of multidisciplinary teams upon arrival of patients with button battery ingestion which dramatically shortens time to removal [5]. Management after extraction of button batteries remains variable, however, depending on the age of the child, location of the battery on removal and endoscopic findings encountered. When complications such as bronchial or tracheo-esophageal fistulas arise, clear guidelines on management are lacking. Here, we present a patient who developed a large broncho-esophageal fistula which was initially managed conservatively with a fenestrated nasogastric tube and ultimately required primary repair with interposition of an intercostal muscle flap. Informed consent statement was obtained for this study. In accordance with the Ann and Robert H. Lurie Institutional Review Board (IRB), this study is exempt from IRB approval.

## CASE REPORT

**Figure 1:**
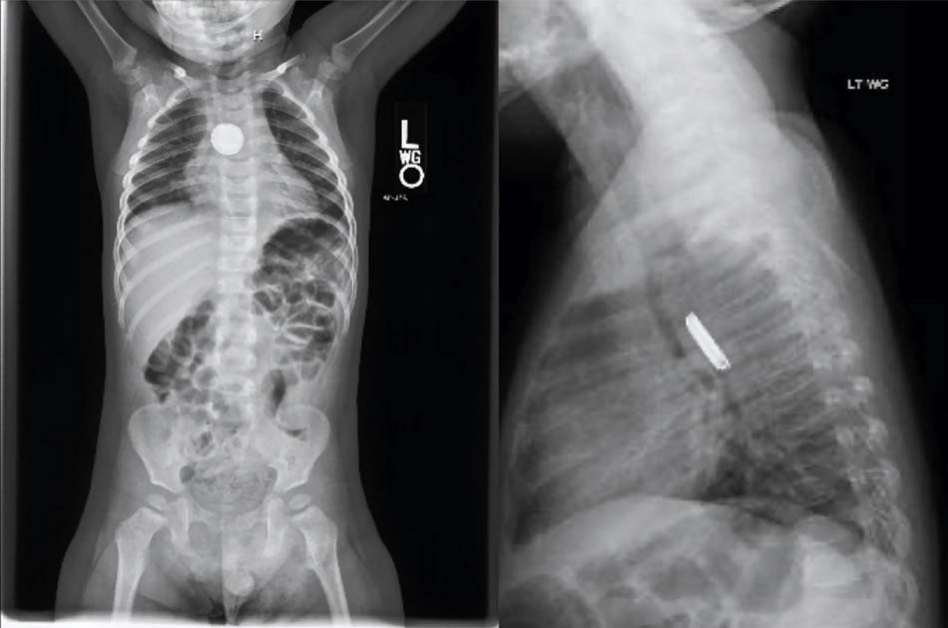
Chest X-ray demonstrated a radiopaque foreign body with halo sign on AP and step-off on lateral view concerning for button battery.

An 18-month-old male presented after his mother noted drooling, non-bilious emesis and a metallic smell to his breath which began 4 days prior. He was febrile to 102.5°F with 100% oxygen saturation on room air. Drooling was noted without stridor. Chest X-ray demonstrated a radiopaque foreign body with halo sign on anteroposterior and step-off on lateral view concerning for button battery (Fig. 1).

**Figure 2:**
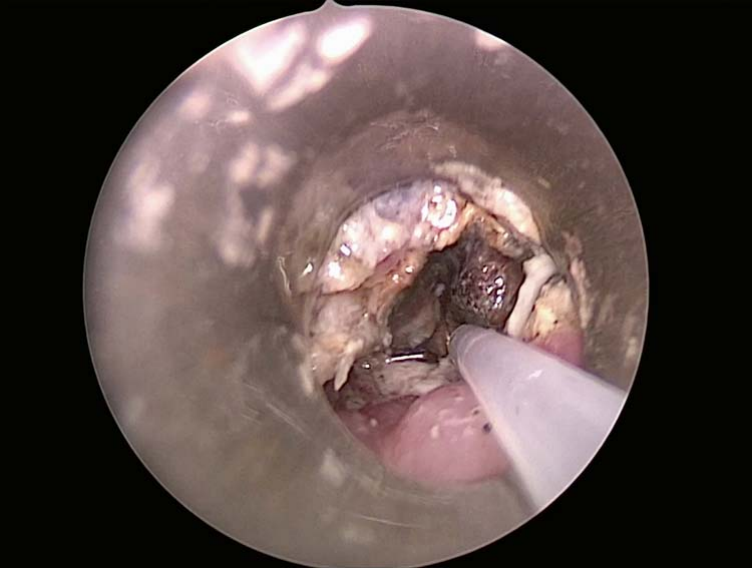
Initial esophagoscopy showing the distal esophagus with near circumferential mucosal injury, sparring the posterior esophageal wall. The injury penetrated through the mucosa into the muscular layer.

The patient was started on intravenous antibiotics and immediately taken to the operating room where micro-direct laryngoscopy, bronchoscopy and esophagoscopy were performed with removal of the 3-V, 20-mm button battery. The distal esophagus had near circumferential mucosal injury, sparring the posterior esophageal wall (Fig. 2). Bronchoscopy also showed superficial injury to the posterior wall of the right mainstem 3 mm in size without clear evidence of broncho-esophageal fistula.

**Figure 3:**
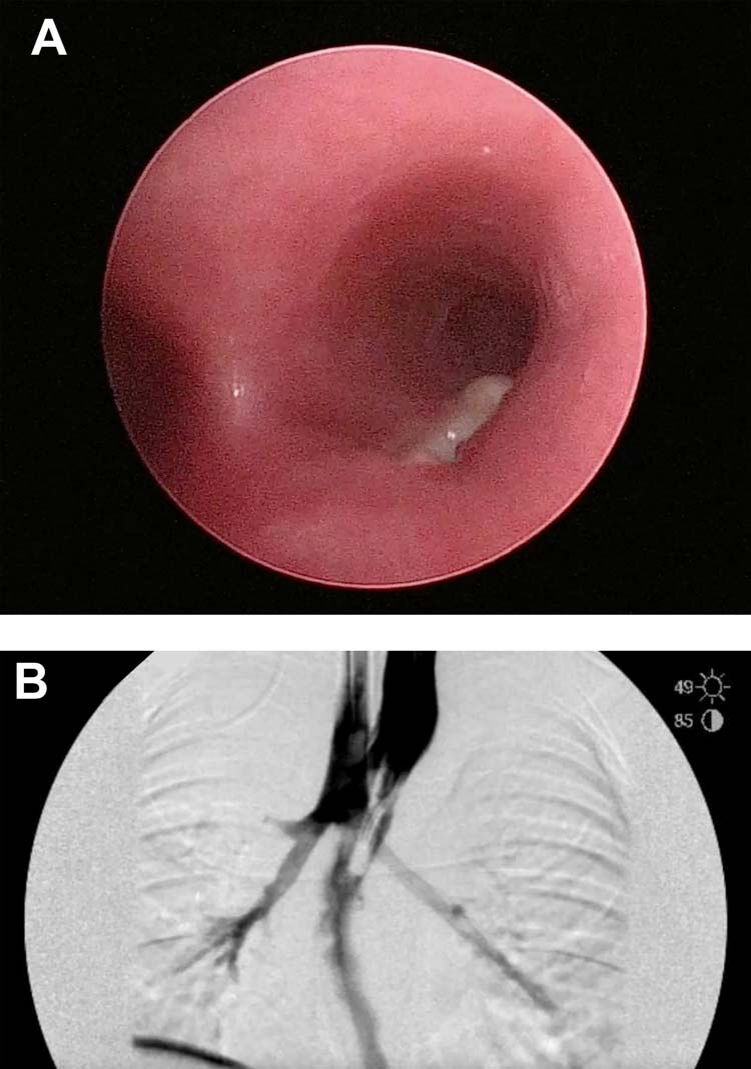
(**A**) Repeat bronchoscopy shows a broncho-esophageal fistula on the posterior wall of the right mainstem bronchus just past the carina. (**B**) Esophagram was performed while in the operating room and clearly shows the fistula to the right mainstem.

Six days later, repeat endoscopy was performed. A 1-cm area consistent with a broncho-esophageal fistula was seen on the posterior wall of the right mainstem bronchus just past the carina (Fig. 3A). Esophagram also clearly showed the fistula (Fig. 3B). A 10 French fenestrated nasogastric tube was placed such that the fenestrations were both above and below the fistula. Feeding jejunostomy tube was also surgically placed. The patient tolerated the procedure well and was able to transition to full feeds.

**Figure 4:**
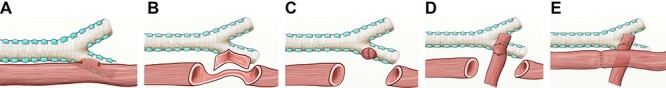
(**A**) Broncho-esophageal fistula shown at posterior right mainstem bronchus. (**B**) The esophagus was transected above and below the area of the fistula with a small rim of muscular esophagus left on the bronchus side of the fistula. (**C**) The mucosa of this short segment was resected and then this area was closed over the fistula track using a series of 5–0 PDS sutures. (**D**) Intercostal muscle flap secured over the fistula repair using 5–0 PDS suture tails that were left in place from the prior closure. (**E**) Completed esophageal anastomosis.

Six weeks later, the patient was again brought to the operating room for final repair of the broncho-esophageal fistula. A new nasogastric tube was placed with lighted stent through the lumen. The patient was positioned in the left lateral decubitus position, skin incision made and soft tissues dissected down to the chest wall. We identified the intercostal muscle in the fifth rib space by transecting toward the sternum and meticulously dissecting the muscle from the ribs while ensuring that we were not hindering the neurovascular bundle. At this point, we used an infrared imaging system (Stryker Endoscopy, San Jose, CA) to highlight the nasogastric tube stent which assisted in the visualization of the proximal esophagus as well as the fistula connection to the right mainstem bronchus (Fig. 4A–E). The esophagus was transected above and below the area of the fistula. The mucosa of this short segment was resected and then this area was closed over the fistula using a series of 5–0 PDS sutures. The aforementioned intercostal muscle flap was secured over the fistula repair using 5–0 PDS suture tails that were left in place from the prior closure. This muscle flap thus buttressed the repair and served as a tissue interposition between the tracheal/bronchial repair and the esophageal anastomosis.

The esophagus was repaired as an end-to-end hand-sewn esophageal anastomosis using 3–0 vicryl sutures. A 16 French chest tube was placed with the tip close to the anastomosis. Esophagram on follow-up 5 days later showed a contained leak that was managed conservatively. The patient advanced to oral feeds but developed a residual esophageal stricture that was managed with serial dilations.

## DISCUSSION

Severe esophageal and airway injuries secondary to button battery ingestions are dangerous and difficult management problems for pediatric surgeons and otolaryngologists. When the button battery lodges in the esophagus, the mucosa bridges the two terminals of the battery completing a circuit [6, 7]. The resulting current generates hydroxide radicals in the tissue leading to liquefactive necrosis. Clinicians must be aware of esophageal, airway and vascular complications associated with these patients in the short term and well after removal of the battery [8, 9]. For example, tracheo-esophageal fistula has been reported 6 days after removal and hemorrhage as many as 18 days after [10]. In stable patients who are higher risk due to young age or larger battery ingestion (>15 mm), repeat films should be obtained as early as 4 days [11]. Repeat endoscopy and further imaging with either CT angiography or MRI should also be considered. Beyond these recommendations, little information exists to guide clinicians.

In this case, the liquefactive necrosis evolved over the first week to a large defect. The decision to wait 6–8 weeks for definitive repair facilitated contracture of the original fistula size and decreased tissue inflammation more amenable to surgical repair. Covered stent placement for tracheo-esophageal fistula after button battery ingestion has been described [12]. In our patient, however, we were concerned about stent migration given the location of the broncho-esophageal fistula immediately below the carina. Some reports exist utilizing endoscopic esophageal vacuum therapy in both children and adults, but these are not tolerated well and often require repeated operating room trips and anesthetic exposures [13, 14].

In this case, the fenestrated nasogastric tube provided excellent secretion control and the feeding jejunostomy tube provided distal enteral access to optimize nutrition while allowing the stomach to be preserved in case a gastric conduit was needed. Using a lighted esophageal stent at the time of repair aided in the visualization of the fistula and adjacent structures. Finally, placing an intercostal muscle flap reinforced the fistula closure and helped prevent recurrence by providing a barrier between the esophagus and bronchus.
